# Enhanced Stem Immobilization Mitigates Leaf Cadmium Accumulation and Modifies PSII Photochemistry in a Tobacco Line with Low Cadmium Accumulation

**DOI:** 10.3390/plants15030483

**Published:** 2026-02-04

**Authors:** Huagang Huang, Jinsong He, Denglu Liu, Haiying Yu, Lu Zhang, Tao Liu

**Affiliations:** 1 College of Resource, Sichuan Agricultural University, 211 Huimin Road, Chengdu 611130, China; m15002827919_1@163.com (J.H.); 13532@sicau.edu.cn (H.Y.); zhangluxqw@163.com (L.Z.); 2 Jianyang Agricultural Technology Extension Center, 220 Xinmin Street, Jianyang 641400, China; liudenglly@163.com

**Keywords:** Cd contamination, tobacco, chemical speciation, subcellular distribution, photosynthesis

## Abstract

Tobacco (*Nicotiana tabacum* L.) has a propensity to accumulate cadmium (Cd), especially in its leaves, which can have a detrimental impact on yield, quality, and product safety. The development of low-accumulation cultivars is a vital mitigation approach; however, the underlying mechanisms remain inadequately understood. In this study, through pot experiments, the physiological mechanisms responsible for the differential Cd accumulation between the low-accumulating tobacco line CF986 and the high-accumulating Yuyan5 were explored. A comprehensive analysis was conducted on the organ-specific Cd distribution, chemical speciation, subcellular compartmentalization, and photosynthetic responses across a gradient of Cd exposure. In comparison with Yuyan5, CF986 accumulated significantly higher amounts of Cd in the roots and stems, but substantially lower amounts in the leaves. Specifically, the Cd content in the leaves of CF986 was only 64.32–68.74% of that in Yuyan5 across different Cd exposure levels. The organ-specific Cd distribution pattern in CF986 followed the order: leaf > stem > root. Moreover, the proportion of Cd partitioned to the leaves was lower in CF986 compared to Yuyan5, while the roots and stems exhibited enhanced Cd retention, with Cd levels in stems reaching up to 2.04 times higher than those in Yuyan5. Analysis of the chemical forms and subcellular distribution of Cd indicated that the mobile Cd fractions in the stems of CF986 were significantly reduced compared to Yuyan5. A larger proportion of Cd was immobilized in the stem cell-wall fraction, which enhanced Cd retention and restricted xylem-mediated transport to the leaves. Cd exposure did not significantly affect the concentration of foliar photosynthetic pigments in CF986; however, it notably inhibited the activity of the photosystem II (PSII) reaction center. At higher Cd levels, the photoprotective thermal dissipation gradually failed, with a decrease of up to 41.36% in Φ_NO_ for CF986 compared to CK under Cd4.0 treatment. This research unveiled a stem barrier mechanism, whereby Cd translocation to the leaves is restricted through chemical and subcellular sequestration in the stem. This mechanism provides a novel perspective on both plant heavy metal allocation and the assurance of crop safety.

## 1. Introduction

Soil contamination by heavy metals, particularly cadmium (Cd), represents a major global environmental challenge with profound implications for food security and human health [1]. Classified by the International Agency for Research on Cancer as one of the most biologically toxic heavy metals, Cd demonstrates high environmental persistence and bioavailability, which facilitate its uptake and accumulation in crops [2]. This is of particular concern for tobacco (*Nicotiana tabacum* L.), an economically important crop cultivated extensively across the globe, which possesses a remarkable capacity for Cd accumulation, especially in its leaves, the primary harvested organ [3]. Given that Cd in tobacco leaves enters the human body directly through smoke inhalation, understanding and reducing Cd accumulation in tobacco are crucial for safeguarding public health.

The behavior of Cd in plants is governed by its chemical speciation and subcellular distribution, which determine its mobility, toxicity, and ultimate accumulation patterns [4]. Cd forms associated with inorganic ions, organic acids, or hydroxyl groups possess high mobility, facilitating their entry into the symplastic pathway. Conversely, when Cd is chelated by pectin, proteins, or insoluble substances like phosphates and oxalates, its translocation capability within the plant is significantly restricted [5]. The subcellular distribution of Cd provides insights into plant tolerance and detoxification mechanisms, among which cell wall fixation and vacuolar compartmentalization play a crucial role. The root cell wall of ZD14 (a low Cd-accumulating cultivar of rice) has a strong ability to fix Cd, leading to low Cd levels in edible tissues [6]. The vacuolar sequestration of Cd in the soluble fractions of K326 (*Nicotiana tabacum*) and *N. rustica* (*Nicotiana rustica*) is one of the significant reasons for the difference in Cd accumulation between the two tobacco genotypes [7].

Beyond concerns regarding accumulation, Cd stress severely impairs plant physiological processes, with photosynthesis being particularly susceptible [8]. Chlorophyll, a sensitive indicator of plant growth under Cd stress, is indispensable for photosynthesis. It plays a vital role in the absorption, transfer, and conversion of absorbed light energy. Consequently, chlorophyll content directly reflects the photosynthetic capacity and efficiency [9]. As an ideal metric of photosynthetic performance, chlorophyll fluorescence is valuable for evaluating light-use efficiency and environmental adaptability. Monitoring photosynthetic pigments and chlorophyll fluorescence parameters offers insights into both the impacts of heavy metal stress on plant growth and the inherent stress tolerance of plants [10]. Understanding how low-Cd-accumulating genotypes maintain photosynthetic function under Cd stress is therefore essential for breeding stress-tolerant cultivars.

Developing low-Cd-accumulating tobacco cultivars represents a sustainable strategy for ensuring product safety while maintaining crop productivity. Previous studies have identified the flue-cured tobacco cultivar CF986 as a low-Cd-accumulating line, Whereas Yuyan5 exhibits a robust capacity for Cd uptake and leaf accumulation [11,12]. However, the physiological and biochemical mechanisms underlying these traits remain to be fully elucidated. This study aims to clarify these mechanisms by comprehensively investigating: (1) organ-specific Cd accumulation and distribution patterns, (2) Cd chemical speciation and subcellular localization, and (3) photosynthetic responses to Cd stress in CF986 compared to Yuyan5. By integrating analyses of Cd behavior with physiological responses, this research provides essential mechanistic insights for the development of strategies to minimize Cd accumulation in tobacco and ensure crop productivity under Cd-contaminated conditions.

## 2. Results

### 2.1. Cadmium (Cd) Accumulation Characteristics in Tobacco Organs

#### 2.1.1. Plant Biomass and Cd Concentration

**Figure 1:**
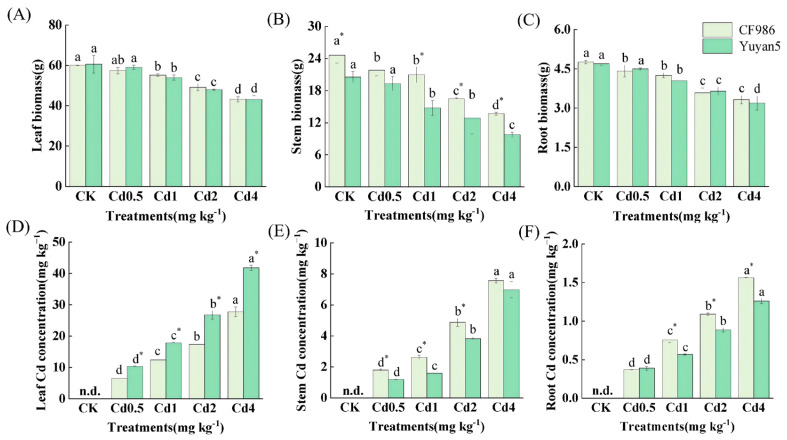
The changes in biomass and Cd concentration in different organs of tobacco under different Cd treatments. (**A**–**C**) biomass of leaf, stem and root. (**D**–**F**) Cd concentration of leaf, stem and root. Different letters indicate significant differences at *p* < 0.05 level of LSD test between different treatments in the same tobacco line. * indicate significant differences at *p* < 0.05 level among two tobacco lines at the same treatment. n.d. means not detected.

The growth of two tobacco lines was significantly inhibited following cadmium treatment (Figure 1A–C). Under varying Cd treatments, no significant differences were observed in leaf or root biomass between CF986 and Yuyan5, whereas the stem biomass of CF986 was significantly higher than that of Yuyan5, which was 1.13–1.42 times. The Cd concentration in each organ of CF986 and Yuyan5 exhibited a significant upward trend after Cd treatment (Figure 1D–F). Under different Cd treatments, the Cd concentration in the stems and roots of CF986 was significantly higher, which was 1.07–1.73 times that of Yuyan5, while the Cd concentration in leaves of CF986 was significantly lower, reaching only 63.53–66.98% of Yuyan5.

#### 2.1.2. Proportion of Cd Accumulation and Distribution

**Figure 2:**
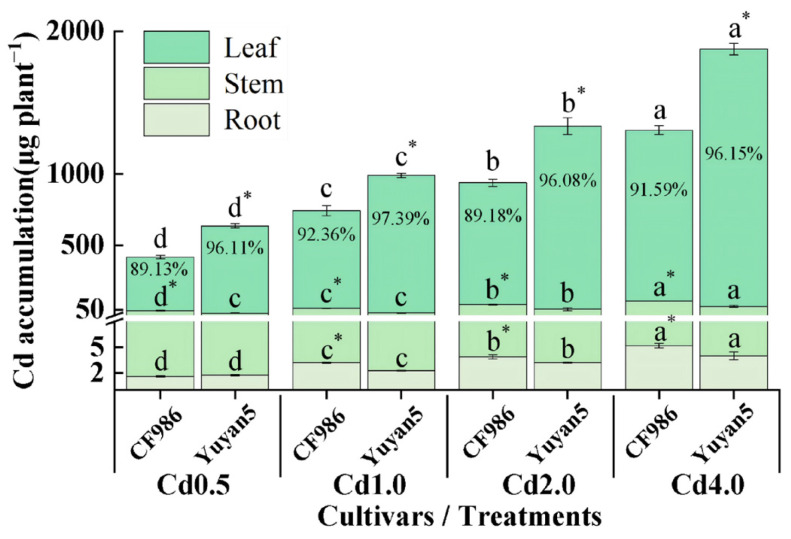
Cd accumulation and distribution characteristics of tobacco organs under different Cd treatments. Different letters indicate significant differences at *p* < 0.05 level of LSD test between different treatments in the same tobacco line. * indicate significant differences at *p* < 0.05 level among two tobacco lines at the same treatment.

A significant increase in Cd accumulation was observed in each organ of the two tobacco lines with the escalation of Cd treatment concentration (Figure 2). The Cd accumulation in the stem of CF986 was significantly higher than that of Yuyan5, ranging from 1.54 to 2.21 times greater. Conversely, the Cd accumulation in leaves of CF986 was significantly lower, being only 64.32% to 68.74% of Yuyan5. The distribution ratio of Cd in each organ was leaf > stem > root, with the proportion of Cd accumulation in leaves exceeding 85%, making it the primary organ for Cd accumulation in tobacco. Under different Cd treatments, the proportion of Cd accumulation in leaves of CF986 was significantly lower than that of Yuyan5, while the proportion in stems was significantly higher, ranging from 0.36 to 2.04 times greater than that of Yuyan5. This suggests that the lower Cd accumulation characteristics of CF986 leaves may be related to the stronger Cd retention ability of its stems.

#### 2.1.3. Bioaccumulation and Translocation Factors

With the increase in Cd treatment concentration, the bioaccumulation factors (BCFs) and translocation factors (TFs) of the two tobacco lines changed significantly (Table 1). The BCF of each organ of CF986 and Yuyan5 showed a downward trend, with both exhibiting a pattern of leaf > stem > root. Under different Cd treatments, the BCF of CF986 leaves was significantly lower than that of Yuyan5, accounting for only 63.45–69.53% of Yuyan5, while the BCF of stems was markedly higher, which was 1.08–1.65 times that of Yuyan5, indicating a stronger capacity for Cd enrichment in stems. The root-to-stem TF in CF986 was generally higher than that in Yuyan5 under the Cd0.5 and Cd1.0 treatments, whereas the stem-to-leaf TF was significantly lower across all Cd treatments. Under identical Cd treatments, the stem-to-leaf TF in CF986 decreased by 38.94–57.93% relative to Yuyan5, suggesting that Cd transport from stems to leaves was effectively restricted in the low-Cd-accumulating line.

### 2.2. Cd Morphology and Subcellular Distribution Characteristics of Tobacco Leaves and Stems

#### 2.2.1. Cd Chemical Forms

**Figure 3:**
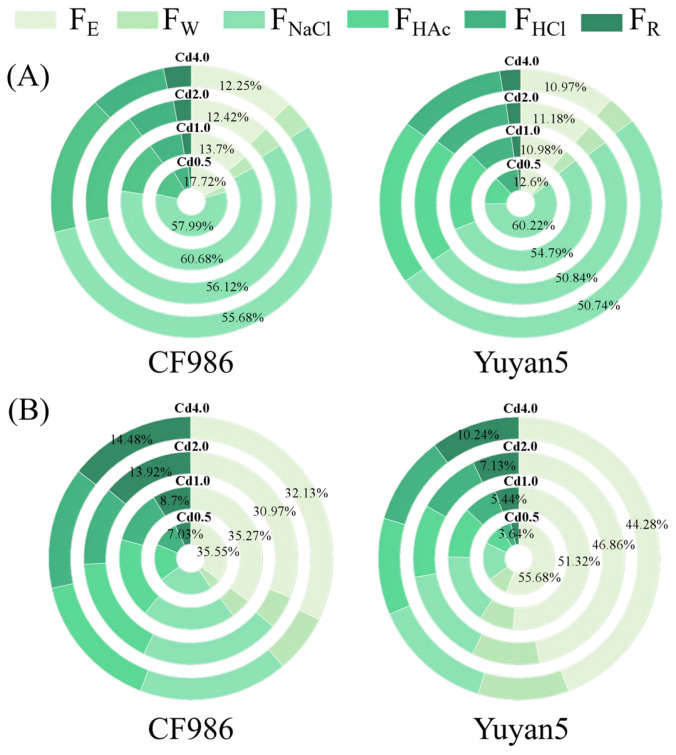
Percentages of different chemical forms of Cd in tobacco cultivars under different Cd treatments. (**A**) Cd Chemical forms of leaf, (**B**) Cd Chemical forms of stem. F_E_, F_W_, F_NaCl_, F_HAc_ and F_HCl_ represent Cd extracted by ethanol, deionized water, NaCl, acetic acid, and HCl, respectively, F_R_ represents Cd in residue.

Under various Cd treatments, the leaves of CF986 and Yuyan5 were predominantly composed of sodium chloride-extractable forms, which constituted 50.74% to 60.68% of the total Cd forms (Figure 3). Notably, the concentrations of ethanol-extractable and water-extractable Cd in CF986 leaves, which exhibit strong mobility, were lower than those in Yuyan5, amounting to only 68.54% to 85.08% of Yuyan5’s levels. Similarly, the concentrations of sodium chloride-extractable, acetic acid-extractable, and hydrochloric acid-extractable Cd with poor mobility also displayed this pattern. There was no significant difference in the residual Cd concentration of leaves.

In the stems, the concentrations of ethanol-extractable and water-extractable Cd in CF986, which are highly mobile, were lower than those in Yuyan5, representing only 60.34% to 85.80% of Yuyan5’s concentrations. Conversely, the concentrations of sodium chloride-extractable, acetic acid-extractable, hydrochloric acid-extractable, and residual Cd in CF986, which have poor mobility, were significantly higher than those in Yuyan5, ranging from 1.45 to 2.82 times the levels found in Yuyan5. This indicates that Cd in the stems of the low-Cd-accumulating tobacco line is present in higher proportions as immobile complexes compared to the high-Cd-accumulating line.

#### 2.2.2. Cd Subcellular Distribution

**Figure 4:**
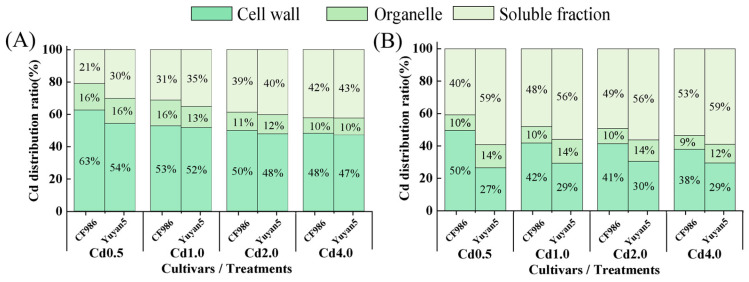
Distribution percentages of Cd in subcellular fractions of tobacco under different Cd treatments. (**A**) Cd subcellular distribution of leaf, (**B**) Cd subcellular distribution of stem.

The leaves of the two tobacco lines were primarily composed of cell wall components, which accounted for 47% to 63% of the total subcellular components, followed by the soluble parts, while the distribution of organelles was the lowest (Figure 4). In contrast, the stems were dominated by soluble components, which constituted 40% to 59% of the total subcellular components, followed by the cell wall, with the lowest distribution ratio for organelles. Additionally, as the Cd treatment concentration increased, the distribution ratio of subcellular components in the stems of the high-Cd-accumulating tobacco line remained relatively unchanged. However, the distribution ratio of leaf cell wall components exhibited a downward trend, and the soluble part showed a corresponding increase. The distribution ratio of the stem cell wall in CF986 was significantly higher than that in Yuyan5, while the subcellular components of the leaves did not differ significantly among the lines. The results suggest that the stem cell wall of the low-Cd-accumulating tobacco line has a greater capacity for Cd binding than that of the high-Cd-accumulating line, thereby restricting its translocation from stem to leaf.

### 2.3. Photosynthetic Pigment Concentration in Tobacco Leaves

**Figure 5:**
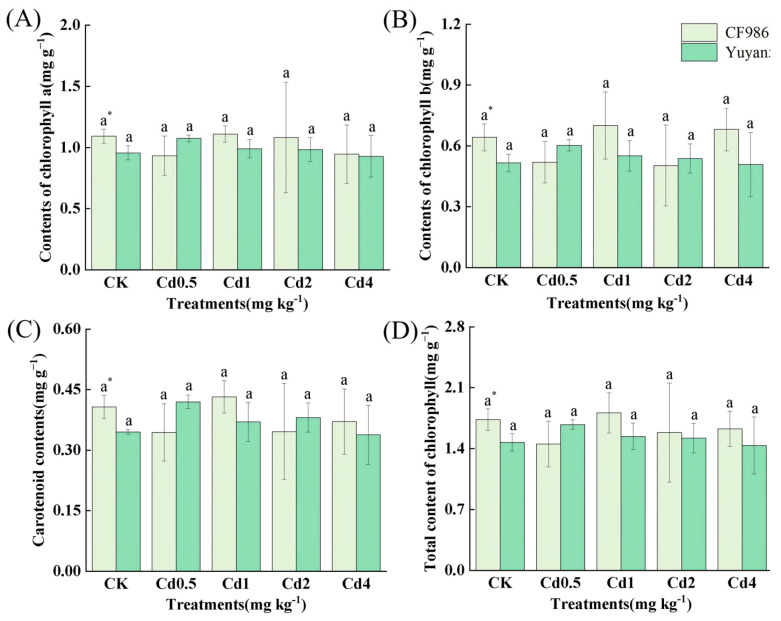
Changes in chlorophyll concentration in tobacco leaves under different Cd treatments. (**A**) Chlorophyll a concentration, (**B**) Chlorophyll b concentration, (**C**) Carotenoid concentration, (**D**) Total concentration of chlorophyll. Different letters indicate significant differences at *p* < 0.05 level of LSD test between different treatments in the same tobacco line. * indicate significant differences at *p* < 0.05 level among two tobacco lines at the same treatment.

It is evident that under control conditions (CK), the concentrations of chlorophyll a, chlorophyll b, carotenoids, and total chlorophyll in the leaves of CF986 were significantly higher than those in Yuyan5. However, under Cd stress, there was no significant difference between the two tobacco lines (Figure 5). Both types of tobacco lines exhibited good tolerance to Cd, as the concentration of photosynthetic pigments in their leaves did not decrease significantly with increasing Cd treatment concentrations.

### 2.4. Chlorophyll Fluorescence Parameters of Tobacco Leaves

From Table 2, it is evident that in comparison with the control (CK), the PSII potential photochemical efficiency (F_v_/F_o_), maximum photochemical efficiency (F_v_/F_m_), and actual photochemical efficiency (Φ_PSII_) of PSII in CF986 and Yuyan5 leaves diminished under various cadmium (Cd) treatments. Conversely, the quantum yield of regulated performance dissipation (Φ_NPQ_) and the quantum yield of non-regulated performance dissipation (Φ_NO_) increased. This suggests that Cd stress impaired the activity of the PSII reaction center in tobacco leaves.

**Figure 6:**
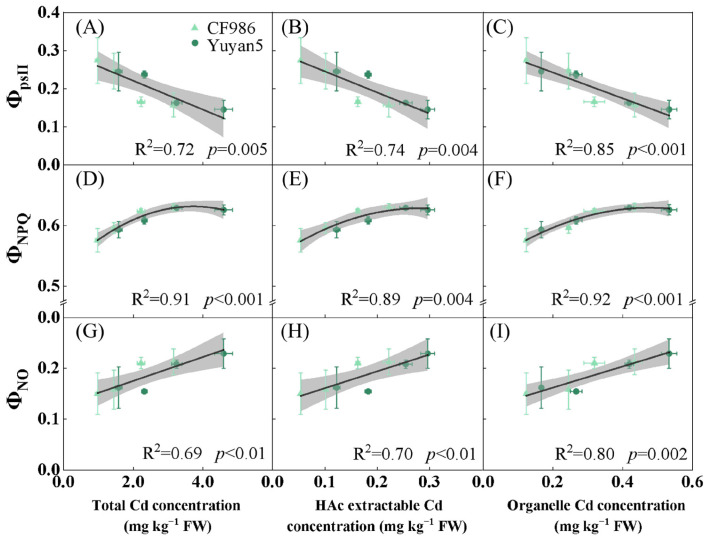
The fitting curves of fluorescence parameters and Cd concentration under different treatments in tobacco leaf. (**A**–**C**) Fitting curves of Φ_psII_ with leaf total Cd concentration, leaf acetic acid extractable Cd concentration, and leaf organelle Cd concentration, respectively. (**D**–**F**) Fitting curves of Φ_NPQ_ with leaf total Cd concentration, leaf acetic acid extractable Cd concentration, and leaf organelle Cd concentration, respectively. (**G**–**I**) Fitting curves of Φ_NO_ with leaf total Cd concentration, leaf acetic acid extractable Cd concentration, and leaf organelle Cd concentration, respectively. The shaded areas represents 95% confidence interval.

The F_v_/F_o_ ratio of CF986 leaves was significantly higher than that of Yuyan5 under different Cd treatments. There was no significant difference in the F_v_/F_m_ ratio between the two lines except for the high concentration Cd treatment, whereas Φ_PSII_, Φ_NPQ_, and Φ_NO_ exhibited differences under lower Cd stress. Under various Cd treatments, the Φ_PSII_, Φ_NPQ_, and Φ_NO_ of CF986 and Yuyan5 leaves correlated well with leaf Cd concentration, acetic acid extractable Cd concentration, and organelle Cd concentration (Figure 6). As the Cd concentration in leaves increased, Φ_PSII_ decreased, Φ_NO_ increased gradually, and Φ_NPQ_ initially rose and then stabilized. The findings indicate that Cd stress diminished the activity of the PSII reaction center, hindered the transfer of photosynthetic electrons within it, and compromised the photosynthetic reaction process and heat dissipation protection mechanism of tobacco leaves.

## 3. Discussion

Cadmium (Cd) is among the most toxic non-essential elements, which can be readily enriched by tobacco shoots. Moreover, tobacco demonstrates a high level of tolerance to Cd [13]. In this research, The significant variation in Cd accumulation capacity between CF986 and Yuyan5 mirrors the findings of Clarke and Brennan [14], who observed similar genotype-dependent variations across sixteen tobacco cultivars in the United States, confirming that selecting low-accumulating genotypes is a globally viable strategy. the low-Cd-accumulating tobacco line CF986 did not display symptoms of Cd poisoning throughout the experiment. Its leaves were able to tolerate Cd while maintaining a relatively high biomass. Even under the Cd stress of 4 mg kg^−1^, the leaf biomass of the low-Cd-accumulating tobacco line decreased only by 4.50% to 28.10% in comparison with the control (CK) treatment.

Tobacco possesses a robust capacity to absorb and accumulate Cd. Generally, tobacco predominantly accumulates Cd in the above-ground tissues, especially in the leaves. It has a strong capacity to transport Cd to the leaves and exhibits characteristics of hyperaccumulators, with more than 80% of absorbed Cd distributed in the leaves [15,16,17]. This finding is consistent with the results of the present study. The proportion of Cd accumulation in the leaves of different tobacco lines with varying Cd accumulation capacities ranged from 89.13% to 97.39%. A similar pattern was also observed in the co-cropping system of cauliflower with *Sedum alfredii*, which indicated that the distribution characteristics of Cd followed the order of leaf > stem > root [18].

The high concentration of Cd in the leaves may be ascribed to the substantial transpiration tension in tobacco leaves. The proportion of Cd in the above-ground parts of tobacco relative to the total Cd concentration in the entire plant was significantly positively correlated with the transpiration rate [19]. It has also been noted that the leaves may not necessarily be the primary organ for Cd accumulation in tobacco. According to Tian [7], under 1 μmol L^−1^ Cd exposure, K326 accumulated Cd in both roots and leaves, Whereas *N. rustica* predominantly accumulated Cd in the root. At high level (50 μmol L^−1^ Cd), Cd was mainly retained in root system of both varieties, indicating a strong root retention capacity under severe Cd stress. In the absence of supplementary cadmium, the Cd concentration in tobacco roots was the highest. Following Cd addition, however, Cd distribution shifted away from root dominance, with greater accumulation observed in aboveground tissues [14]. This study yielded similar findings, confirming that the leaves were the primary site of Cd accumulation.

Bioaccumulation factors (BCFs) reflect the enrichment of heavy metals in different plant organs relative to soil concentrations, while translocation factors (TFs) evaluate the transport capacity of heavy metals between organs within plants [20]. It is widely recognized that plants with BCF and TF values greater than 1 have the potential for Cd hyperaccumulation, enabling upward translocation of heavy metals and thereby facilitating their removal from soil through methods such as harvesting [21]. The shoot BCFs and TFs of *Lantana camara* L. ranged from 1.78 to 4.78 and from 1.34 to 4.90, respectively [22]. In this study, the BCF and TF values of stems and leaves of the two tobacco lines were greater than 1, indicating that tobacco has a strong capacity to accumulate and transport Cd upward. The stem BCF and TF values of the low-Cd-accumulating line CF986 were significantly higher than those of Yuyan5, whereas the leaf values were lower, which can be attributed to the stronger Cd retention capacity in the stems of CF986.

The chemical speciation of Cd in plants significantly influences the intracellular Cd ion concentrations, thereby affecting both phytotoxicity and mobility. This variation in speciation contributes to differences in Cd uptake and translocation among plant species and genotypes [23,24]. Using sequential extraction methods with specific chemical reagents, Cd in plant tissues can be fractionated into different forms, each exhibiting distinct mobility and toxicity. For instance, the ethanol- and deionized water-extractable forms, which are primarily associated with inorganic ions and organic acids, are highly mobile and biologically active. In contrast, other forms—bound to pectin, proteins, metal acid salts, or oxalates—exhibit limited mobility and lower toxicity [25,26].

Previous studies on Brassica species have consistently shown that low-Cd-accumulating cultivars preferentially retain Cd in low-mobility chemical forms, particularly NaCl- and acetic acid-extractable fractions, compared with high-Cd-accumulating cultivars. In Chinese cabbage and Chinese flowering cabbage, such cultivars exhibited elevated NaCl-extractable Cd in roots or whole plants, accompanied by reduced proportions of more mobile water- and ethanol-extractable forms [27,28]. In this study, the stems of CF986 primarily contained ethanol-extractable Cd, followed by sodium chloride-extractable Cd. Compared to the high-Cd-accumulating line, Cd in the stems of the low-Cd-accumulating line was predominantly present in low-mobility forms, thereby reducing Cd mobility within the plant This finding partially diverges from earlier reports, possibly due to crop-specific differences in Cd uptake and storage mechanisms [29]. In the leaves of both tobacco lines, Cd was mainly present in the NaCl-extractable form, suggesting an association with pectin and proteins, likely sequestered in the cell wall. This compartmentalization may help mitigate Cd toxicity, explaining why tobacco leaves accumulate higher Cd concentrations yet exhibit fewer toxicity symptoms than roots and stems [30].

Subcellular distribution plays a critical role in Cd accumulation and translocation. Mechanisms such as cell wall binding and vacuolar compartmentalization in the soluble fraction are key to limiting Cd translocation to shoots [31]. The capacity for Cd retention in the cell wall varies among species. For example, in the non-hyperaccumulating ecotype of *Sedum alfredii*, most Cd in root cells is sequestered in the cell wall, thereby reducing its mobility [32]. Similarly, the cadmium-safe rice line D62B retains more Cd in root cell walls, thus mitigating Cd toxicity [33]. In this study, the stem cell walls of the low-Cd-accumulating tobacco line CF986 contained significantly higher Cd concentrations than those of the high-Cd-accumulating line, indicating enhanced Cd retention in the stem apoplast, which likely restricts Cd transfer to leaves and accounts for the lower Cd accumulation observed in CF986 leaves. Our observation that Cd is compartmentalized in the cell wall aligns with the foundational work of Wagner and Yeargan [34] on American tobacco cultivars, where tissue fractionation revealed that intracellular sequestration plays a pivotal role in controlling Cd distribution.

Additionally, vacuolar sequestration in the soluble fraction represents another important mechanism for Cd detoxification, with genotypic variations widely observed [35]. For instance, low-Cd-accumulating varieties of pepper and rice demonstrate a greater capacity for vacuolar Cd compartmentalization in roots [36,37]. Compared with *N. tabacum*, the higher root Cd retention in *N. rustica* has been attributed to enhanced cell wall binding, stronger Casparian strip barriers, and less efficient xylem loading [38].

Chlorophyll serves as a key indicator for assessing photosynthetic performance and plays an essential role in light energy capture and transfer. Carotenoids absorb residual light energy, quench singlet oxygen, prevent membrane lipid peroxidation, and protect chlorophyll and photosynthetic function [39]. Cd^2+^ can disrupt chloroplast structure, inhibit the synthesis of photosynthetic pigments, and replace magnesium ions in chlorophyll molecules, thereby inactivating them [40]. In this study, the concentration of photosynthetic pigments in the leaves of both tobacco lines showed no significant change under different Cd treatments, suggesting the activation of a defense mechanism in tobacco to preserve photosynthetic pigments under Cd stress.

Chlorophyll fluorescence parameters revealed that the tobacco leaves initiated a thermal dissipation protection mechanism during the photosynthetic process. Chlorophyll fluorescence, which primarily originates from chlorophyll a in antenna pigments, serves as an effective probe for monitoring photosystem II (PSII) activity in functional leaves, reflecting the absorption, transfer, dissipation, and distribution of light energy by PSII. The F_v_/F_m_ ratio is highly sensitive to environmental stress and reflects the extent of damage to the PSII complex, serving as a valuable indicator of plant stress tolerance [41]. In this study, F_v_/F_m_ values in both tobacco lines remained relatively stable with increasing Cd concentrations, except under the Cd4.0 treatment, where a significant decline was observed. This indicates that a protective mechanism was active under low Cd stress but failed under severe Cd stress-a trend consistent with observations in *Cosmos bipinnatus* Cav. exposed to Cd treatment [42].

F_v_/F_o_ represents the potential photochemical efficiency of PSII in plant leaves. Under Cd treatment, the F_v_/F_o_ in tobacco leaves gradually decreased. Notably, the low-Cd-accumulating line CF986 maintained higher F_v_/F_o_ values than Yuyan5, indicating a stronger potential photochemical capacity. The actual photochemical efficiency (Φ_psII_) was used to evaluate the photochemical state of PSII under light conditions. Φ_psII_ was significantly reduced in both tobacco lines under Cd stress. When Cd^2+^ enters the chloroplasst, it deposits on the thylakoid membranes and binds to sulfhydryl groups in membrane proteins [43], causing protein denaturation and loss of function. It may also replace Ca^2+^ in the PSII reaction center, thereby inhibiting PSII photochemical activity [44].

Photosynthetic electron transfer relies on the coordinated interaction between chlorophyll and electron carrier proteins. It is speculated that Cd disrupts the structure of electron transfer proteins in tobacco leaves, impairs energy transfer in antenna systems, reduces electron excitation in reaction centers, and ultimately inhibits quantum efficiency and electron transport [45]. Non-photochemical quenching (NPQ) acts as a self-protective mechanism for the photosynthetic apparatus. Enhanced non-photochemical energy dissipation helps to disperse excess excitation energy, mitigating environmental stress and protecting the PSII reaction center from photodamage [46]. Among NPQ components, Φ_NPQ_ reflects the regulated energy dissipation yield at PSII and serves as an important indicator of photoprotection, whereas Φ_NO_ represents the non-regulated energy dissipation yield and indicates the extent of photodamage. In this study, Φ_NO_ increased progressively with increasing Cd concentrations in both tobacco lines. Compared to the control, Cd4.0 treatment increased Φ_NO_ by 38.31% and 41.36%, respectively, indicating that the incident light intensity exceeded the tolerable threshold for the plants. Without adequate photochemical mitigation, PSII would suffer damage. Under Cd4.0 treatment, Φ_NPQ_ increased by 11.50% and 5.21%, respectively, compared to the control. At this stage, PSII experienced photoinhibition, suggesting that the photoprotective mechanisms mediated by energy dissipation in tobacco leaves had gradually deteriorated.

**Figure 7:**
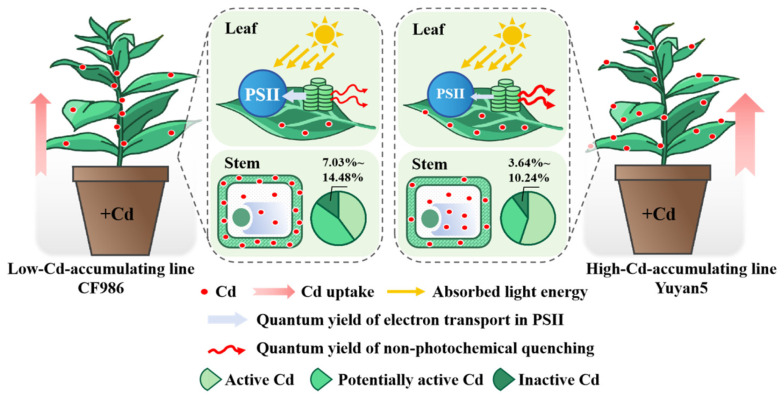
Proposed schema for the difference pattern in Cd accumulation and photosynthetic characteristics between the two tobacco lines.

Based on the above findings, the following conclusions can be drawn (Figure 7). Significant differences in leaf Cd accumulation were observed between the low-Cd-accumulating tobacco line CF986 and the high-Cd-accumulating line Yuyan5, which primarily stemmed from the markedly distinct Cd retention capacity in the stems. Specifically, Cd was present predominantly in a low-mobility chemical forms, and was sequestered within stem cell walls at the subcellular level. Together, these characteristics effectively restrict Cd translocation to the leaves. Furthermore, under Cd stress, the low-Cd-accumulating line exhibited stronger PSII activity and sustained less photodamage compared to the high-Cd-accumulating line.

## 4. Materials and Methods

### 4.1. Experimental Material

Soil Material: Soil samples were collected from Shuangbai Village in Puyang Town, Dujiangyan City. The basic physical and chemical properties were as follows: pH 6.45, organic matter 29.5 g kg^−1^, total nitrogen 0.71 g kg^−1^, alkali-hydrolyzable nitrogen 92.5 mg kg^−1^, available phosphorus 15.0 mg kg^−1^, available potassium 151.0 mg kg^−1^, and the soil total Cd concentration was not detected.

Plant Material: In a previous study, two tobacco (*Nicotiana tabacum* L.) lines were identified: CF986 as a low-Cd-accumulating line, and Yuyan5 as a high-Cd-accumulating line [12]. Both were flue-cured tobacco lines with similar growth periods, provided by Liangshan Tobacco Company of Sichuan Province.

Fertilizer Material: The fertilizers used were analytically pure reagents: ammonium nitrate (N 35%), potassium dihydrogen phosphate (P_2_O_5_ 52%, K_2_O 34%), and potassium sulfate (K_2_O 54%).

### 4.2. Experimental Design

Cd treatments were designed at 0, 0.5, 1.0, 2.0, 4.0 mg Cd kg^−1^, supplied as Cd chloride hydrate (CdCl_2_ 2.5H_2_O). Each treatment was replicated three times, resulting in a total of 30 pots arranged in a completely randomized design. The application rates of nitrogen (N), phosphorus (P_2_O_5_), and potassium (K_2_O) fertilizers were 90, 105, and 300 mg kg^−1^ soil, respectively. After drying, crushing, sieving, and mixing the soil, each pot (15 L) was filled with 15 kg of soil. Before transplanting, phosphate fertilizer, 50% of the nitrogen fertilizer, and 40% of the potassium fertilizer were applied to the soil as basal fertilizer in aqueous solution, and Cd was added and aged for 30 days (Table 3). The remaining nitrogen and potassium fertilizers were top-dressed at 15 days (10% potassium) and 40 days (50% nitrogen, 50% potassium) after transplanting. Tobacco seedlings with identical growth conditions were selected for transplanting, with one plant per pot. Natural light (The photoperiod was approximately 10–12 h, and the light intensity at the canopy typically ranged between 800 and 1200 μmol photons m^−2^ s^−1^ during clear days) was used. To minimize potential spatial heterogeneity in light conditions, pots were randomly repositioned at regular intervals, and all treatments were maintained under the same environmental conditions, ensuring comparable light exposure across treatments. The experiment was conducted in a net room with rainproof facilities in the teaching and research park of Sichuan Agricultural University.

### 4.3. Sample Collection and Preparation

Plants were harvested at the peak of vegetative growth (55 days after transplanting). The collected plant samples were thoroughly washed with tap water to remove soil particles. To remove surface-adsorbed Cd, the roots were immersed in 20 mmol L^−1^ Na_2_-EDTA solution (Kelong Chemical Co., Ltd., Chengdu, China) for 15 min, followed by rinsing three times with deionized water. Subsequently, the plants were separated into roots, stems, and leaves. Samples intended for biomass and total Cd determination were oven-dried at 105 °C for 30 min to deactivate enzymes, and then dried at 75 °C to a constant weight. The dried samples were ground into fine powder and passed through a 0.25 mm (60-mesh) sieve. Fresh leaves used for photosynthetic pigment and chlorophyll fluorescence measurements were analyzed immediately. The remaining fresh leaves intended for the determination of Cd chemical forms and subcellular distribution were flash-frozen in liquid nitrogen and stored at −80 °C in an ultra-low temperature freezer (Thermo Fisher Scientific, Waltham, MA, USA).

### 4.4. Methods of Determination

#### 4.4.1. Determination of Cd Concentration in Soil and Plants

The total Cd concentration in soil was determined by digesting 0.1 g of air-dried soil with a mixed acid solution of HNO_3_-HClO_4_-HF (5:1:1, *v*/*v*/*v*) at 180 °C on a hot plate until the digestion was complete. The available Cd concentration in soil was extracted using the DTPA extraction method [47]. Specifically, 10 g of air-dried soil was placed in a polyethylene bottle, and 20 mL of extraction solution containing 0.005 M DTPA (Aladdin Biochemical Technology Co., Ltd., Shanghai, China), 0.01 M CaCl_2_, and 0.1 M triethanolamine (Sinopharm Chemical Reagent Co., Ltd., Shanghai, China) adjusted to pH 7.3 was added. The mixture was shaken at 180 rpm for 2 h at room temperature (25 °C) and then filtered. For plant samples, 0.2 g of dried plant powder was digested using a mixture of HNO_3_ and HClO_4_ (4:1, *v*/*v*) at 160 °C. The Cd concentrations in the digested and extracted solutions were determined using an atomic absorption spectrophotometer (PinAAcle900, Perkin Elmer, Waltham, MA, USA).

#### 4.4.2. Isolation of Subcellular Fractions for Cd Analysis

Subcellular fractions were processed following a previously established method with slight modifications [48]. Specifically, 0.5 g of fresh tissue was homogenized in 10 mL of cold extraction buffer (50 mM Tris-HCl, 250 mM sucrose, and 1.0 mM dithioerythritol, pH 7.5; Kelong Chemical Co., Ltd., Chengdu, China) using a pre-cooled mortar and pestle. The homogenate was centrifuged at 4000 rpm for 15 min at 4 °C using a refrigerated centrifuge (TGL-16M, Xiangyi Centrifuge Instrument Co., Ltd., Changsha, China). The precipitate was designated as the cell wall fraction (F1). The supernatant was further centrifuged at 16,000 rpm for 30 min at 4 °C. The resulting precipitate was the organelle fraction (F2), and the final supernatant was the soluble fraction (F3). All steps were performed at 4 °C. The F1 and F2 fractions were digested with HNO_3_-HClO_4_ (4:1, *v*/*v*), while the F3 fraction was used directly for measurement. Cd concentrations in all fractions were determined by atomic absorption spectrophotometry as described above.

#### 4.4.3. Extraction of Cd Chemical Forms in Plants

Cd chemical forms were extracted using a sequential extraction method with the following solvents: 80% (*v*/*v*) ethanol (F_E_), deionized water (F_W_), 1 mol L^−1^ NaCl (F_NaCl_), 2% (*v*/*v*) acetic acid (F_HAc_), and 0.6 mol L^−1^ HCl (F_HCl_). Specifically, approximately 0.3 g of homogenized fresh leaf tissue was transferred into a centrifuge tube containing 10 mL of the first extraction solution. The mixture was shaken at 25 °C for 22 h and then centrifuged at 5000× *g* for 10 min using a centrifuge (TGL-16M, Xiangyi Centrifuge Instrument Co., Ltd., Changsha, China). The supernatant was collected, and the extraction process was repeated once for each solvent to ensure complete extraction. This procedure was performed sequentially for all five extractants. The final solid residue (F_R_) was dried at 75 °C to a constant weight and digested with HNO_3_ using a microwave digestion system (TOPEX+, PreeKem Scientific Instruments Co., Ltd., Shanghai, China). The digested solution was diluted to a final volume of 10 mL with deionized water. Cd concentrations in all supernatants and the digested residue solution were determined using inductively coupled plasma mass spectrometry (ICP-MS 8900, Agilent Technologies, Santa Clara, CA, USA) [49].

#### 4.4.4. Determination of Photosynthetic Pigment Concentration

The collected fresh leaves were wiped clean, veins removed, and cut into small pieces. Approximately 0.1 g of the leaf samples was weighed and submerged in 20 mL of a mixed extraction solution containing acetone and anhydrous ethanol (1:1, *v*/*v*; Kelong Chemical Co., Ltd., Chengdu, China). The samples were kept in the dark at room temperature for approximately 24 h until the leaf tissues turned completely white. The absorbance of the extract was measured at 663 nm, 645 nm, and 470 nm using a UV-visible spectrophotometer (UV-2600, Shimadzu, Kyoto, Japan). The concentrations of chlorophyll a (Chl a), chlorophyll b (Chl b), and carotenoids (Car) were calculated based on the formulas described by Xiong [50].

#### 4.4.5. Determination of Chlorophyll Fluorescence Parameters

Chlorophyll fluorescence parameters were measured using an Imaging-PAM modulated fluorescence imaging system (IMAGING-PAM, Walz GmbH, Effeltrich, Germany). Before measurement, the leaves were dark-adapted for 30 min. The minimal fluorescence (F_o_) and maximal fluorescence (F_m_) were recorded to calculate the PSII potential photochemical efficiency (F_v_/F_o_) and maximum photochemical efficiency (F_v_/F_m_). Subsequently, the leaves were exposed to actinic light to determine the actual photochemical efficiency (Φ_PSII_), quantum yield of regulated energy dissipation (Φ_NPQ_), and quantum yield of non-regulated energy dissipation (Φ_NO_).

### 4.5. Calculation of Relevant Parameters

The bioaccumulation factor (BCF) was calculated as the ratio of the Cd concentration in plant tissues (roots, stems, and leaves) to that in the soil. Conversely, the translocation factor (TF) was calculated as the ratio of the Cd concentration in stems or leaves to that in the roots [51,52]. Cd accumulation in various organs of tobacco was determined by multiplying the Cd concentration by the biomass of the plant tissues (roots, stems, and leaves). The Cd distribution ratio in various organs of tobacco was calculated as the ratio of Cd accumulation in the plant tissues (roots, stems, and leaves) to that in the entire plant. The total chlorophyll concentration was determined by adding the chlorophyll a concentration to the chlorophyll b concentration.

### 4.6. Statistical Analysis

All data were presented as the mean ± standard deviation (SD) of the three replicates. Statistical analysis was performed using the SPSS Statistics software 27 with a two-way ANOVA. Differences were considered significant at a *p*-value of less than 0.05, estimated using the least significant difference (LSD) test. All tables and figures were generated using Excel 2013 and Origin (Version 9.0).

## 5. Conclusions

This study demonstrated that the low-leaf-cadmium trait in tobacco line CF986 was regulated by a pronounced “stem barrier” mechanism. This barrier operated through two synergistic processes: the chemical immobilization of cadmium into low-mobility forms and its active sequestration within stem cell walls. Together, these processes effectively restrict Cd translocation via the xylem, leading to significantly reduced accumulation in leaves compared to the high-Cd-accumulating line Yuyan5, and further leading to differences in photosynthetic characteristics between the two lines. These findings revealed a previously unrecognized stem-based cadmium retention strategy in tobacco, providing both a physiological basis for reducing Cd accumulation in leaves and a promising target for molecular breeding in leafy crops. Future studies should investigate the molecular mechanisms underlying stem Cd immobilization to support the development of low-Cd-accumulating cultivars.

## Figures and Tables

**Table 1:** The data come from the average of three replicates, columns and error bars represent means ± SE (*n*  =  3). Different letters indicate significant difference (*p* < 0.05) among different treatments. * indicates significant difference between the two tobacco lines (*p* < 0.05).

Treatments	BCF						TF			
	Root		Stem		Leaf		Root-Stem		Stem-Leaf	
	CF986	Yuyan5	CF986	Yuyan5	CF986	Yuyan5	CF986	Yuyan5	CF986	Yuyan5
Cd0.5	0.83 a	0.87 a	4.03 a *	2.67 a	14.69 a	23.15 a *	4.88 a *	3.07 c	3.65 ab	8.67 b *
Cd1.0	0.77 b *	0.57 b	2.66 b *	1.61 c	12.60 b	18.13 b *	3.48 c *	2.81 d	4.74 a	11.24 a *
Cd2.0	0.58 c *	0.47 c	2.58 b *	2.03 b	9.22 c	14.20 c *	4.47 b	4.32 b	3.57 b	7.00 c *
Cd4.0	0.38 d *	0.31 d	1.83 c	1.69 c	6.74 d	10.14 d *	4.84 a	5.54 a *	3.67 b	6.01 d *

**Table 2:** Leaf chlorophyll fluorescence parameters of tobacco leaves under different Cd treatments at peak stage. Different letters indicate significant differences at *p* < 0.05 level of LSD test between different treatments in the same tobacco line. * indicate significant differences at *p* < 0.05 level among two tobacco lines at the same treatment.

Treatments	Cultivars	F_v_/F_o_	F_v_/F_m_	Φ_psII_	Φ_NPQ_	Φ_NO_
CK	CF986	2.06 a *	0.819 a	0.281 a *	0.565 c	0.154 bc
	Yuyan5	1.80 a	0.811 a	0.243 a	0.595 c *	0.162 c *
Cd0.5	CF986	2.01 a*	0.819 a	0.274 a *	0.576 bc	0.150 c
	Yuyan5	1.82 a	0.820 a	0.245 a	0.593 c *	0.162 c *
Cd1.0	CF986	1.93 b*	0.816 a	0.246 b	0.596 b	0.158 b
	Yuyan5	1.76 ab	0.809 a	0.237 a	0.609 b *	0.154 c
Cd2.0	CF986	1.80 c*	0.817 a	0.166 c	0.624 a	0.210 a
	Yuyan5	1.73 b	0.805 ab	0.163 b	0.629 a	0.208 b
Cd4.0	CF986	1.73 d*	0.804 b *	0.157 c	0.630 a	0.213 a
	Yuyan5	1.61 c	0.784 b	0.145 c	0.626 a	0.229 a

**Table 3:** Characteristics of soil Cd pollution after homogenization. The data come from the average of six replicates, columns and error bars represent means ± SE (*n*  =  6). Different letters indicate significant differences at *p* < 0.05 level of LSD test between different treatments in the same tobacco line.

Indexes	Treatments				
	CK	Cd0.5	Cd1	Cd2	Cd4
Total Cd (mg kg^−1^)	n.d.	0.45 d	0.99 c	1.88 b	4.12 a
Available Cd (mg kg^−1^)	n.d.	0.23 d	0.53 c	0.93 b	1.91 a

## Data Availability

This manuscript does not report data generation or analysis. Data are available from the authors upon request.
